# LONGITUDINAL ASSOCIATIONS OF LONELINESS AND FRAILTY IN COMMUNITY DWELLING OLDER ADULTS

**DOI:** 10.1093/geroni/igad104.3457

**Published:** 2023-12-21

**Authors:** Chava Pollak, Joe Verghese, Aron Buchman, Helena Blumen

**Affiliations:** Albert Einstein College of Medicine, Bronx, New York, United States; Albert Einstein College of Medicine, Bronx, New York, United States; Rush University, Chicago, Illinois, United States; Albert Einstein College of Medicine, Bronx, New York, United States

## Abstract

Loneliness is highly prevalent among older adults and is associated with poor health outcomes. This study examined longitudinal associations between loneliness and frailty and the influence of other objective and subjective social measures on these associations. We analyzed data from 1,3111 older adults without dementia at baseline from the Rush Memory and Aging Project (mean age 78.3 ±7.5 years, 72.5% female). Loneliness was assessed using the de Jong Gierveld Loneliness Scale. Frailty was defined using a 39-item cumulative deficit frailty index. Linear mixed effects models were used to examine the association of baseline loneliness and change in frailty over time adjusted for baseline age, gender, education, depressive symptoms, global cognition, social network, marital status, social support, and social, cognitive, and physical activity. Loneliness and frailty both increased significantly over a mean follow-up period of 4.6 years. Baseline loneliness was associated with an additional increase in frailty by 0.3 deficits per year compared to those who were not lonely (p< 0.001, CI 0.2, 0.5). The 0.3 increase in frailty per 1-point increase in loneliness equaled a 27% increase in frailty per year. Loneliness is associated with accelerated frailty and should be considered in the design of interventions to prevent frailty and promote healthy aging.

